# Prevalence and genotypic identification of *Cryptosporidium* in free-ranging and farm-raised donkeys (*Equus asinus asinus*) in Xinjiang, China

**DOI:** 10.1051/parasite/2020042

**Published:** 2020-06-25

**Authors:** Wen Wang, Zhenjie Zhang, Ying Zhang, Aiyun Zhao, Bo Jing, Longxian Zhang, Pengtao Liu, Meng Qi, Wei Zhao

**Affiliations:** 1 College of Animal Science, Tarim University Alar Xinjiang 843300 P.R. China; 2 College of Animal Science and Veterinary Medicine, Henan Agricultural University Zhengzhou Henan 450002 P. R. China; 3 College of Life Sciences, Tarim University Alar Xinjiang 843300 P.R. China; 4 Department of Parasitology, Wenzhou Medical University Wenzhou Zhejiang Province 325035 P.R. China

**Keywords:** *Cryptosporidium*, Donkey, Genotyping, Zoonotic potential

## Abstract

The prevalence and zoonotic potential of *Cryptosporidium* in donkeys is poorly understood. Here, 680 fecal specimens were collected from 178 free-ranging and 502 farmed donkeys in Xinjiang, China. *Cryptosporidium* was identified using PCR amplification of the small subunit of ribosomal DNA. *Cryptosporidium-*positive isolates were subtyped using PCR analysis of the 60 kDa glycoprotein gene (*gp60*). The overall prevalence of *Cryptosporidium* was 2.4% (16/680), with 3.2% (16/502) in farmed donkeys and 0% (0/178) in free-ranging donkeys. *Cryptosporidium hominis* (*n* = 13), *C. parvum* (*n* = 1) and *Cryptosporidium* horse genotype (*n* = 2) were identified. The *C. hominis* isolates belonged to two subtypes, IkA16 (*n* = 9) and IkA16G1 (*n* = 4). The subtype of *C. parvum* was IIdA15G1, whereas the two *Cryptosporidium* horse genotype isolates were of subtype VIaA15G4. The predominance of *C. hominis* in donkeys suggests that these animals are infected through human contact.

## Abstract (fr)

La prévalence et le potentiel zoonotique de *Cryptosporidium* chez les ânes sont mal connus. Dans cet article, 680 spécimens fécaux ont été prélevés de 178 ânes en liberté et 502 ânes en élevage dans le Xinjiang, en Chine. Les *Cryptosporidium* ont été identifiés en utilisant une amplification par PCR de la petite sous-unité de l’ADN ribosomique. Les isolats positifs pour *Cryptosporidium* ont été sous-typés en utilisant une analyse PCR du gène de la glycoprotéine 60 kDa (gp60). La prévalence globale de *Cryptosporidium* était de 2,4 % (16/680), avec 3,2 % (16/502) chez les ânes d’élevage et 0 % (0/178) chez les ânes en liberté. *Cryptosporidium hominis* (*n* = 13), *C. parvum* (*n* = 1) et *Cryptosporidium* génotype chevalin (*n* = 2) ont été identifiés. Les isolats de *C. hominis* appartenaient à deux sous-types, IkA16 (*n* = 9) et IkA16G1 (*n* = 4). Le sous-type de *C. parvum* était IIdA15G1, tandis que les deux isolats de *Cryptosporidium* génotype chevalin étaient du sous-type VIaA15G4. La prédominance de *C. hominis* chez les ânes suggère que ces animaux sont infectés par contact humain.

## Introduction

*Cryptosporidium* is an important zoonotic parasitic pathogen that causes diarrhea in humans and various animal species [19]. To date, at least 39 species and more than 70 genotypes of *Cryptosporidium* have been described [8, 13]. Among them, *C. hominis* and *C. parvum* are the most common species identified in humans [6, 15]. Previous studies indicate that *C. hominis* and *C. parvum* are the dominant species that infect horses and donkeys [8, 14].

The zoonotic nature of various *Cryptosporidium* species implies that public health may be affected by infected animals [19]. Contact with farmed animals has been identified as a risk factor for disease in case–control studies of sporadic human cryptosporidiosis [7]. Additionally, several outbreaks involving veterinary and farm school students have been documented, including children and students infected with *C. parvum* after visiting open farms and outdoor adventure farms in England [5, 12]. Moreover, an Italian study reported a foal at a racecourse with severe diarrhea caused by *C. parvum* infection, and the subsequent infection of six students with the same subtype of *C. parvum* after visiting the racecourse, resulting in diarrhea or abdominal pain [11].

The donkey has been used as a working animal for at least 5000 years. China has the largest number of donkeys, at 11 million, and they are used principally as pack animals or for draught work in transport or agriculture. Some donkeys are raised for milk or meat, as well as for making Ejiao, a traditional Chinese medicine product. To our knowledge, only two reports have been published about *Cryptosporidium* infection of donkeys in China [14, 17]. The present study aimed to determine the prevalence and zoonotic potential of *Cryptosporidium* in free-ranging and scale-farmed donkeys in Xinjiang, China.

## Materials and methods

### Ethics statement

The research protocol was reviewed and approved by the Research Ethics Committee of Tarim University. Before collecting fecal specimens, we contacted the managers of donkey farms or donkey owners, and obtained their permission to have their animals involved. No animals were injured during this procedure.

### Collection of fecal specimens

From May 2016 to December 2018, 680 fresh fecal specimens (approximately 50 g per specimen) were collected from 178 free-ranging donkeys at five locations and 502 farmed donkeys from 18 farms in 12 cities of Xinjiang, China (Table 1). All fecal specimens were collected from the ground immediately after defecation using a sterile disposable latex glove. The number of collected specimens accounted for 10%–30% of the adult or young donkeys at each farm and all free-ranging donkeys in the countryside. All specimens were transported to the laboratory in a cooler with ice packs, and stored at 4 °C until processing. The ages of the adult and young donkeys were ≥1 year and <1 year old, respectively. None of the animals showed any clinical symptoms at the time of sampling.

**Table 1:** Prevalence, species/genotypes and subtypes of *Cryptosporidium* in donkeys in Xinjiang, China.

Feeding pattern	Collection site	Age (year)	No. positive/No. samples (%)	*Cryptosporidium* species, subtype (*n*)
Farmed				
	Alaer (Farm 1)	<1	0/54	/
	Barkol (Farm 2)	<1	2/11 (18.2)	*C.* horse genotype (2), VIaA15G4 (2)
	Bohu (Farm 3)	≥1	0/27	/
	Bohu (Farm 4)	≥1	2/34 (5.9)	*C. hominis* (2), IkA16 (2)
	Bohu (Farm 5)	<1	1/18 (5.6)	*C. hominis* (1), IkA16 (1)
	Gongliu (Farm 6)	<1	0/21	/
	Huocheng (Farm 7)	<1	0/20	/
	Karakax (Farm 8)	<1	5/41 (12.2)	*C. hominis* (5), IkA16 (3), IkA16G1 (2)
	Karakax (Farm 9)	<1	0/47	/
	Khorgas (Farm 10)	Unclear	0/20	/
	Pishan (Farm 11)	Unclear	0/26	/
	Pishan (Farm 12)	Unclear	0/15	/
	Qitai (Farm 13)	≥1	0/16	/
	Turpan (Farm 14)	<1	0/17	/
	Yopurga (Farm 15)	<1	3/12 (25.0)	*C. hominis* (3), IkA16 (3)
		≥1	1/24 (4.2)	*C. parvum* (1), IIdA15G1 (1)
	Yopurga (Farm 16)	<1	0/10	/
		≥1	0/44	/
	Yopurga (Farm 17)	<1	2/13 (15.4)	*C. hominis* (2), IkA16G1 (2)
	Yuli (Farm 18)	<1	0/32	/
	Subtotal		16/502 (3.4)	C. hominis (13), IkA16 (9), IkA16G1 (4); C. parvum (1), IIdA15G1 (1); Cryptosporidium horse genotype, VIaA15G4 (2)
Free-ranging				
	Akqi (Location 1)	≥1	0/11	/
	Barkol (Location 2)	≥1	0/21	/
	Pishan (Location 3)	≥1	0/48	/
	Yecheng (Location 4)	≥1	0/64	/
	Zepu (Location 5)	≥1	0/34	/
	Subtotal		0/178	/
Total		<1	13/296 (4.4)	*C. hominis* (11), IkA16 (7), IkA16G1 (4); *Cryptosporidium* horse genotype (2), VIaA15G4 (2)
		≥1	3/323 (0.9)	*C. hominis* (2), IkA16 (2); *C. parvum* (1), IIdA15G1 (1)
		Unclear	0/61	*/*

### DNA extraction

All the fecal specimens were sieved through an 8.0-cm-diameter sieve with a pore size of 45 μm, and the filtrates were concentrated by centrifugation at 1500× *g* for 10 min, then genomic DNA was extracted from approximately 200 mg of precipitates using an E.Z.N.A. stool DNA kit (Omega Biotek Inc., Norcross, GA, USA), according to the manufacturer’s instructions. A total of 200 μL of extracted DNA from each specimen was transferred to Eppendorf tubes and stored at −20 °C until PCR amplification.

### Genotyping and subtyping of *Cryptosporidium*

*Cryptosporidium* spp. were identified using nested PCR amplification of a partial small subunit of ribosomal DNA (SSU rDNA) gene fragment of ~830 bp using primers previously described by Xiao et al. [21]. All *Cryptosporidium*-positive isolates were further subtyped using nested PCR amplification of an ~850 bp fragment of the gene encoding a 60-kDa glycoprotein (*gp60*) using primers previously described by Alves et al. [2]. All PCR amplifications included positive controls (chicken-derived *C. bailey* DNA) and negative controls (dH_2_O).

### DNA sequencing and analysis

All nested PCR-positive products were sent for sequencing by GENEWIZ (Suzhou, China). The accuracy of the sequencing data was confirmed by sequencing the PCR products using both the forward and the reverse primer. Species and genotypes of *Cryptosporidium* were identified by comparing nucleotide sequences with each other and with published GenBank sequences using the Basic Local Alignment Search Tool (BLAST) (http://blast.ncbi.nlm.nih.gov/Blast.cgi) and ClustalX2.11 (http://www.clustal.org/).

### Statistical analysis

Data entry and analysis were performed using SPSS 19.0 software (IBM Corp., Armonk, NY, USA). Prevalence was calculated according to feeding pattern and age (young *vs.* adult donkey). Categorical variables were expressed as numbers of cases (percentages) and frequencies were compared using chi square tests with *p* < 0.05 considered statistically significant.

### Nucleotide sequence accession numbers

Representative sequences of *Cryptosporidium* were deposited in GenBank under accession numbers MK731970 to MK731972 (SSU rRNA) and MK731973 to MK731976 (gp60).

## Results and discussion

Sixteen fecal specimens were positive for *Cryptosporidium* from six farms in four cities with an overall infection rate of 2.4% (16/680), which is similar to that previously reported for donkeys in Algeria (1.6%, 2/124) [16]. The infection rate of *Cryptosporidium* in farmed donkeys was 3.2% (16/502), whereas none of the free-ranging donkeys harbored *Cryptosporidium* infection (Table 1). The infection rate of *Cryptosporidium* in young donkeys (4.4%, 13/296) was higher than that in adults (0.9%, 3/323), and this difference was statistically significant (χ^2^ = 7.36, *p* < 0.01). In fact, it is not only in donkeys that young animals are more likely to carry *Cryptosporidium* infections, this is true for most host species. There are a great many studies, especially in cattle, which clearly indicate a much higher prevalence of *Cryptosporidium* in young animals [1, 4]. These data suggest that age may be a risk factor for *Cryptosporidium* infection in animals including donkeys. Moreover, *Cryptosporidium* infections can be transmitted amongst animals on farms because of group feeding habits and shared feeding environments. The present study did not include any young free-ranging donkeys, only young farmed donkeys, which might have skewed the infection rate findings.

To date, three *Cryptosporidium* species (*C. parvum*, *C. muris*, and *C. hominis*) and only one genotype (*Cryptosporidium* horse genotype) have been identified in donkeys [14, 16, 17]. In the present study, 16 *Cryptosporidium-*positive specimens underwent successful sequencing of the SSU rDNA gene. Three species/genotype were identified, including *C. hominis* (*n* = 13), *C. parvum* (*n* = 1), and the *Cryptosporidium* horse genotype (*n* = 2) (Table 1).

*Cryptosporidium hominis* had the highest frequency (81.3%, 13/16) in this study, and was detected on six *Cryptosporidium-*positive farms from four areas (Table 1). *C. hominis* is widely considered a human-specific *Cryptosporidium* species, but also commonly infects nonhuman primates, horses, and donkeys, which appear to be typical hosts [8, 14, 16]. Most *C. hominis* isolates from horses and donkeys have been identified as divergent subtypes of the host-adapted Ik subtype family, including IkA15G1, IkA20G1, IkA16, and IkA16G1 [14]. In the present study, two subtypes were identified, IkA16 (*n* = 9) and IkA16G1 (*n* = 4), with IkA16 as the dominant subtype (Table 1). To date, these have only been identified in horses and donkeys, indicating that they are host-adapted *C. hominis* subtypes with limited public health significance.

*Cryptosporidium parvum* has been detected in donkeys from Algeria and China [14, 16]. In the present study, *C. parvum* was detected in an adult farmed donkey, as subtype IIdA15G1 (Table 1), which differs from the previously reported *C. parvum* subtype IIdA19G1 in donkeys from Shandong and Henan, China [14]. The IIdA15G1 subtype has been identified in humans and a variety of animals, being particularly common in cattle and various rodents in China [9]. The IIdA15G1 subtype of *C. parvum* in a donkey was likely to have been transmitted from humans or other animals, particularly cattle and rodents, but the real source of infection and transmission require further analysis.

The *Cryptosporidium* horse genotype has been identified in donkeys in China [14]. In the present study, the *Cryptosporidium* horse genotype was detected in two young farmed donkeys with the same subtype, VIaA15G4 (Table 1). This subtype has been detected in China, Italy, and the Czech Republic [3, 10, 14, 18, 20]. Currently, only two subtype families have been identified in the *Cryptosporidium* horse genotype: VIa in animals and VIb in humans and hedgehogs [14]. The host specificity of the VIa and VIb subtype families is not completely understood because of limited data, so further investigation is needed to elucidate the source of the *Cryptosporidium* horse genotype infection and the cross-species transmission potential of this genotype in donkeys, other animals, and humans in China.

## Conclusions

This study demonstrated the occurrence of *Cryptosporidium* infection in donkeys in Xinjiang, China. Three species or genotype of *Cryptosporidium* were identified, including *C. parvum*, *C. hominis*, and the *Cryptosporidium* horse genotype; all of these have previously been detected in humans. Future epidemiologic studies of *Cryptosporidium* should preferentially focus on donkeys to understand the true epidemiology of *Cryptosporidium* and its transmission dynamics in China.

## Conflict of interest

The authors declare that they have no conflict of interest.
